# Social activities and depressive symptoms among migrant middle-aged and older adults in China: a network analysis

**DOI:** 10.3389/fpsyg.2024.1376180

**Published:** 2024-06-13

**Authors:** Qian Liu, Yuanyuan Wu, Chang Yu, Yaolin Pei

**Affiliations:** ^1^School of Public Administration, Hunan Normal University, Changsha, China; ^2^School of Humanities and Social Science, Xi'an Jiaotong University, Xi'an, China; ^3^College of Arts and Sciences, University at Buffalo, State University of New York, Buffalo, NY, United States; ^4^Rory Meyers College of Nursing, New York University, New York City, NY, United States

**Keywords:** depressive symptom, social activity, migrant middle-aged and older adults, network analysis, Chinese

## Abstract

**Background:**

This study investigated the central symptom within the depression network and examined the relationship between social activities and depressive symptoms among migrant middle-aged and older adults in China.

**Methods:**

We analyzed data from 1,926 migrants aged 45 and older, derived from the 2018 China Health and Retirement Longitudinal Study (CHARLS). Using network analysis, we identified the central depressive symptom and assessed the association between various social activities and depressive symptoms.

**Results:**

Network analysis revealed that depressed mood was the most central symptom. Regarding mitigation of depressive symptoms, informal social activities predominantly influenced positive emotions and somatic symptoms. Formal activities were mainly revealed through positive emotions. Solitary activities were manifested primarily through positive emotions and somatic symptoms. In addition, informal and solitary activities showed a stronger correlation with the alleviation of depressive symptoms compared to formal activities.

**Conclusion:**

The findings underscore the importance of addressing depressed mood in treating depression among migrant middle-aged and older adults. Recognizing the differential impacts of various social activities can aid in the development of customized prevention and intervention strategies aimed at enhancing the mental well-being of this demographic in China.

## 1 Introduction

### 1.1 Migration and depression

In China, the “hukou” system operates as a household registration mechanism, functioning akin to a domestic passport (Chan et al., 1999). This system categorizes individuals based on their place of origin, assigning them a “hukou” status that is either “agricultural” (rural) or “non-agricultural” (urban). Chan (2013) delineates two distinct types of internal migration within China considering this system. The first type is characterized by migration with “local” residency rights, commonly referred to as hukou migration. Conversely, the second type involves migration without hukou residency rights and is termed non-hukou migration. In this study, migration is specifically defined as non-hukou migration, concentrating on middle-aged and older adults categorized as migrants. Specifically, the migrant population in this study refers to individuals whose hukou location does not consistent with their actual place of residence.

With the progression of urbanization, the middle-aged and older migrant population in China has surfaced as a pivotal component driving internal migration. Data from 2018 indicates that this group amounted to 13.04 million, constituting 5.3% of the entire migrant population. This demographic segment has witnessed a substantial increase since the turn of the century, rising from 5.03 million in 2000 to 13.04 million by 2015. Furthermore, this growth trajectory represents an average annual growth rate of 6.6%, underscoring the rapid expansion of this population segment (National Health Commission of the People’s Republic of China, 2018). Previous studies have identified family factors as significant determinants in the migration decisions of older adults in China (Lu and Qin, 2014). For example, the primary motivation for migration among urban older individuals is to care for grandchildren, while rural older adults predominantly migrate in search of employment opportunities (Cheng et al., 2019). Additionally, Gao et al. (2021) suggest that individuals in poorer health may be more inclined to migrate, seeking the support and security that coresident adult children can offer.

Studies examining the association between international migration and health showed that immigrants often exhibit better health outcomes than non-immigrants (Halliday and Kimmitt, 2008). This is attributed to two hypotheses: the “healthy migrant effect,” indicating that healthier individuals with better access to resources are more likely to migrate (Chiswick et al., 2008), and the “salmon bias hypothesis,” suggesting that aging immigrants with declining health may return to their origin (Abraido-Lanza et al., 1999). Both hypotheses have been applied to China’s internal migration (Lu and Qin, 2014), but limited research has focused on the aging migrant population in China. This group potentially faces mental health challenges due to the restrictive hukou system, raising various structural and social barriers to restrict migrants’ access to social welfare and entitlement attached to an urban hukou (Zhang et al., 2021) and the disruption of social circles. The resulting lack of emotional support can amplify depression, leading to a higher prevalence of mental health issues among migrant older individuals compared to urban aging groups (Liu et al., 2020). Therefore, protecting the mental health of migrant middle-aged and older adults should be an important public health priority.

To our best knowledge, the majority of the existing Chinese migrant studies treated depression as a whole using scales that summed individual item responses to generate a total score, implying that depressive symptoms are viewed as interchangeable presentations of the same disorder (Guo et al., 2017). However, empirical evidence revealed that individual depressive symptoms were associated with a variety of adverse outcomes, risk factors, and neurological mechanisms (Fried and Nesse, 2014). Additionally, clinical evidence suggests that the central depressive symptoms are more likely to activate other symptoms and, thus, play a significant role in causing the onset of a syndrome and maintaining it (Berlim et al., 2020). For instance, animal and human models imply that restricted sleep causes depression and anxiety symptoms, and hopelessness predicts suicide ideation (Baglioni et al., 2011). Accordingly, targeting central symptoms with biological or psychosocial interventions rather than peripheral symptoms can be highly effective (Borsboom and Cramer, 2013). This indicates that a latent factor model of depression may not fully capture the complexity of the depressive phenomenon. Hence, this study aims to employ network analysis to explore the depression structure and identify the central symptoms of depression in Chinese migrant middle-aged and older adults.

### 1.2 Social activity and depression

Social engagement, defined as the performance of meaningful social roles for leisure or productive activities, could protect against depression. Strong evidence suggests that activity participation is effective in decreasing depression. Namely, participation in social activities may protect individuals against depression by stimulating multiple bodily systems and reinforcing life-long patterns of attachment (James et al., 2011). However, older adults often lack opportunities for social activities due to diminished physical and motor functionality. Consequently, they are more likely to feel lonely and isolated, which in turn is associated with depression (Hao et al., 2017).

Although social activities are associated with depressive symptoms, the direction and strength of the association depend on the type of social activities. Activity theory of aging, a leading framework for understanding the link between activities and mental health, categorizes social and leisure activity participation into three broad types: informal activities involve socializing with familiar individuals such as relatives, friends, or neighbors; formal activities encompass participation in structured groups and organizations; and solitary activities, such as reading, watching television, and pursuing various hobbies (Lemon et al., 1972). These solitary activities are characterized by the ability to be performed either alone or with limited social interaction (Li and Tang, 2022). Besides, this theory asserts that informal activity is more effective than formal activity. In contrast, solitary activity is the least effective of the three activity types for helping older people improve life satisfaction (Lemon et al., 1972). However, scholars have not agreed on the order of these three types of social activities. For instance, as claimed by Ritchey et al. (2001), informal social activity was related to more aspects of well-being than formal and solitary activity. However, Longino and Kart (1982) suggested that informal activities are positively associated with life satisfaction. Other studies have suggested that solitary activities are not associated with life satisfaction and that formal activities have a negative effect on life satisfaction. Additional Asian studies also approved that informal activities might have a greater impact on geriatric depression than formal social activities in older people (Wang et al., 2020).

In some studies, however, participation in formal activities such as religious organizations and community activities is more beneficial to mental health than participation in other types of social activities in Western countries (Hao et al., 2017). The underlying reason may be that the social significance of social activities varies across cultural contexts. Specifically, previous evidence showed that Asians tend to have a family-oriented culture, and Asian older adults tend to participate in fewer formal activities than Western older adults (Chiao et al., 2011). Moreover, although Longino and Kart (1982) pointed out that there was no association between solitary activities and late-life satisfaction, solitary activities such as internet use could relieve depression among middle-aged and older migrant adults in China (Liu et al., 2020). In detail, older migrants may use the Internet to reconcile their loneliness by participating in activities, interacting with others, and strengthening connections with members of society.

Previous research has focused on exploring the different effects of informal, formal, and solitary activities on depression in older adults at a holistic level. However, the specific pathways of association between these three types of activities on depressive symptoms among migrant middle-aged and older adults are still unclear, and it is necessary to utilize a network analysis approach to deeply analyze how these three types of activities are associated with depressive symptoms and which activities are most effective in alleviating depressive symptoms. Besides, another primary purpose of this study is to understand the current patterns of social activities and depression among older adults in China’s migration context and, therefore, explore the associations between individual depressive symptoms and types of social activities at different levels from various aspects. Older individuals need to examine such patterns to select appropriate life behaviors and thus target them to improve their mental health.

### 1.3 Network approach for investigation

Network analysis, a set of procedures based on the modeling of dynamical systems, provides a visual depiction of the complex associations among symptoms (Beard et al., 2016). Different from the traditional way of treating mental disorders as potential entities that cause symptoms, the network approach emphasizes the connection between the symptoms themselves (Borsboom and Cramer, 2013). Nodes and edges in the network are the elements of the syndrome. The higher the activation degree of the node, the thicker the edge represents the more serious mental disorder (Fried et al., 2017). Analyzing depressive symptoms from the perspective of network analysis allows us to exceed the current average level of symptoms and understand which symptoms may be particularly central to the depression experience (Borsboom, 2017). A growing body of literature utilizes network analysis to detect the depression structure among older individuals. For example, “sadness,” “empty,” “hopeless,” “pessimistic,” “suicidal thought,” and “lassitude” were identified as core symptoms of late-life depression in Korea (Kim et al., 2021). Whereas “death wishes,” “depressed mood,” “loss of interest,” and “pessimism” are identified as the central symptoms of depression among European older adults (Murri et al., 2020). Pan and Liu (2021) claimed that “I felt sad” and “I could not get going” were the two most important depressive symptoms among Chinese widowed older adults. Given the high prevalence of depression in the Chinese immigrant population, it is essential to identify the structure of depressive symptoms in this population.

Additionally, a detailed pathway indicating how factors affect the symptoms of a psychological mental disorder could be clarified when they are involved in the network (Robinaugh et al., 2014). In this study, from the network analysis perspective, depressive symptoms and social activities could be regarded as causally interacting entities, with each subset of the network of factors being different but interdependent. The most important advantage of network analysis is its feasibility of identifying core nodes and associated pathways for timely intervention (Hofmann et al., 2016). Thus, examining the association between different types of social activities and depressive symptoms helps develop tailored prevention and intervention strategies that aim to improve the mental health of migrant middle-aged and older adults in China.

### 1.4 Current study

This study employs data from the 2018 China Health and Retirement Longitudinal Study (CHARLS), a nationally representative sample, to examine the symptom central to the network of depression and the relationship between social activities and depressive symptoms among migrant middle-aged and older adults in China through network analysis. Specifically, building on existing research that identified depression among this demographic, this study utilizes a network analysis approach to pinpoint the symptom central to the network of depression in migrant middle-aged and older adults. Besides, we explored the specific ways in which different types of social activities are associated with individual depressive symptoms among the migrant middle-aged and older adults in China.

## 2 Methods

### 2.1 Data and sample

The China Health and Retirement Longitudinal Study (CHARLS) is a nationally representative sample of the middle- and old-age population (45+) in China. Data for this study come from the 2018 wave of the China Health and Retirement Longitudinal Study (CHARLS). Underpinned by the multistage stratified probability proportional to size (PPS) sampling technique, the CHARLS research team surveys middle- and old-aged community-dwelling residents aged 45 years old and above from 450 villages in 150 counties of 28 provinces in China (Zhao et al., 2020). The baseline CHARLS survey was carried out in 2011 and involved 17,708 individuals, with biannual follow-ups conducted in 2013 and 2015. So far, the latest version of the survey has been conducted in 2018. Data Research Topic covered a wide array of domains, including demographic characteristics, family structure, health status and functioning, health care and insurance, work, retirement and pension, income, and expenditure and assets.

We utilized the Hukou system, which regulates population movement within China, to identify a subsample of middle-aged and older migrants residing in various villages. Initially, 2,346 participants were selected. However, those who did not provide responses to questions about depressive symptoms were excluded, resulting in a final sample size of 1,926 for this research.

### 2.2 Measures

#### 2.2.1 Depressive symptoms

Depressive symptoms were measured by the 10-item Center for Epidemiologic Studies-Depression scale (CES-D). The CES-D10 reported good psychometric properties in older Chinese people. Examples of items concluded “I was bothered by things that do not usually bother me” and “I had trouble keeping my mind on what I was doing.” Respondents reported how often they felt this way during the past week (0 = rarely or none of the time; 1 = not too much time; 2 = sometimes or half the time; 3 = most of the time). Each item was coded with a specific label from D01-D10. Since D05 and D08 were positively scored, we coded them in reverse. Higher scores indicated more severe depressive symptoms. The Appendix A provides full information about this scale.

#### 2.2.2 Informal activities

Informal activities were measured with two items. Participants responded whether they took part in the following activities in the last month: interacted with friends; played mah-jong (“麻将” in Chinese), playing chess, playing cards, or going to a community club. For each question, the options for participants to select were 0 = no and 1 = yes. Each item was coded with a specific label from I01 to I02.

#### 2.2.3 Formal activities

Formal activities were measured with two items. Respondents reported whether they took part in the following activities in the last month: took part in a community-related organization; done voluntary or charity work with non-paying time and energy, including providing help to family, friends, or neighbors and caring for a sick or disabled adult. For each question, the options for participants to select were 0 = no and 1 = yes. Each item was coded with a specific label from F01 and F02.

#### 2.2.4 Solitary activities

Solitary activities were measured with two items. Participants reported whether they participated in the following activities in the last month: went to a sport, such as dancing, keeping fit, and playing Qigong; used the Internet. For each question, the options for participants to select were 0 = no and 1 = yes. Each item was coded with a specific label from S01 and S02.

#### 2.2.5 Health variables

Health variables contained physical disability and chronic illness. Physical disability was measured using the Activities of Daily Living (ADL) and Instrumental Activities of Daily Living (IADL) scales. The ADL scale assesses a person’s ability to dress, bathe, eat, go to the toilet, get in or out of bed, and control urination and defecation. The IADL scale assesses an older person’s capability to do household chores, prepare hot meals, shop, phone, take medications, and manage money. We dichotomized the disability status into two groups (no functional problems = 0, has at least one limitation = 1). Chronic illness was assessed by 14 items: hypertension, dyslipidemia, high blood sugar, cancer or malignant tumor, lung disease, liver disease, heart attack; stroke; kidney disease; digestive diseases; emotional, nervous, or psychiatric problems; memory-related disease; arthritis or rheumatism, and asthma. Respondents who had any of these diseases were defined as having one or more chronic illnesses, while those who responded did not have all chronic illnesses were defined as not having a chronic illness.

#### 2.2.6 Sociodemographic variables

Sociodemographic variables included age, gender (male = 1, female = 0), marital status (married = 1, unmarried = 0), hukou (agricultural = 1, non-agricultural = 0), religious faith (having = 1, not having = 0), education (in years), living with children or not (yes = 1, no = 0), household *per capita* consumption expenditure (RMB).

### 2.3 Data analysis strategy

Age, gender, education, marital status, hukou, religious faith, living with children, household *per capita* consumption, chronic disease, and physical disability were used as control variables in this research. In this research, all statistical analyses were performed with R software. We estimated and visualized the structure of depressive symptoms by using the R package of qgraph. This package can automatically compute the appropriate correlations according to the types of variables (Maccallum and Bryant, 2020). We chose Spearman’s rank correlation to estimate regularized partial correlation networks (Epskamp and Fried, 2018) due to the ordinal nature of the item. To control for false positives and create a sparse and interpretable network, the GLASSO procedure used the graphical “least absolute shrinkage and selection operator” (LASSO) regularization with extended Bayesian information criterion (EBIC) model selection (Epskamp and Fried, 2018; Maccallum and Bryant, 2020). We used a regularization approach with tuning parameter γ (specifying the level of sparsity) set to 0.5 (Foygel and Drton, 2010) to compute potential spurious relationships. A psychopathology network is displayed graphically as a set of symptoms (nodes), with nodes connecting through a set of relations (edges). However, recent literature suggested that non-regularized networks might be preferable in some cases, especially for large sample sizes. Since this is not the case for our sample, we presented the non-regularized partial correlation network in Supplementary Figures S1, S2.

First, we computed one network of depressive symptoms, wherein nodes represent observed items and edges represent partial correlation coefficients between two items after conditioning for all other variables in the migrant middle-aged and older adults’ dataset (Epskamp et al., 2018). Blue edges indicate positive correlations, and red edges indicate negative correlations. The stronger the correlation between items, the thicker an edge is. Centrality is commonly used to assess the importance of nodes. We calculated three statistical indices of centrality to identify which items were the core depressive symptoms: Strength (the sum of the absolute values of the number of edges connecting to a node), Betweenness (the number of items a node acts as a bridge along the shortest path between two other nodes) and Closeness (average distance from the node to all other nodes in the network) (Beard et al., 2016).

Second, the relationships among the items of depression, informal activities, formal activities, and solitary activities were investigated using network analysis as well. We estimated one association network, including depressive symptoms, informal activities, formal activities, and solitary activities in the migrant middle-aged and older adults’ dataset, in which nodes and edges also represent observed items and partial correlation coefficients between two items, respectively. In contrast to previous studies that used network analyses in the field of psychopathology, we focused on comparing specific edges rather than centrality measures when networks including social factors were computed. These networks are composed of depressive items and several nodes about social participation factors, making the interpretation of centrality measures indicative of centrality to the network of symptoms problematic (Burger et al., 2020).

Finally, we tested the edge-weight accuracy and centrality stability of these networks by using the bootnet package in R software. The case-dropping bootstrap method was run with 5,000 samples. The correlation stability coefficient (CS-coefficient) was calculated to show the stability of node orders based on the degree index. The edge-weight accuracy was computed using a non-parametric bootstrapping method. Plots of edge-weight accuracy and centrality stability of each network were shown in Supplementary Figures S3–S6.

## 3 Results

### 3.1 Sample characteristics

Table 1 outlines the characteristics of the study participants. The average age of respondents is 60.19 years, with a standard deviation of 9.58 years, and ranges from 45 to 95 years. Participants typically had 6.16 years of education, with a standard deviation of 4.76 years, and education levels varied from none to 22 years. The average household *per capita* consumption expenditure was 13,725.06 yuan, with a considerable variation, as indicated by a standard deviation of 22,235.95 yuan, and expenditures ranged from zero to 420,620 yuan. Just over half of the participants were female and did not have any chronic illnesses. The majority were married, held an agricultural hukou, lacked religious affiliations, lived without children, and had no functional impairments.

**Table 1:** The characteristics of the sample (*n* = 1926).

Variable	Mean (%)	*SD*	Range
Age	60.19	9.58	45–95
Gender			
Male	48.08%		
Female	51.92%		
Marital status			
Married	88.79%		
Unmarried	11.21%		
Hukou			
Agricultural	63.97%		
Non-agricultural	36.03%		
Religious faith			
Having	8.98%		
Not having	91.02%		
Education (in years)	6.16	4.76	0–22
Living with children			
Yes	24.92%		
No	75.08%		
Household per capital consumption expenditure (RMB)	13725.06	22235.95	0–420,620
Chronic disease			
One or more chronic illness	44.96%		
No	55.04%		
Physical disability			
One or more functioning limitations	13.60%		
No functional problems	86.40%		

Table 2 shows the mean, standard deviation (SD), min value, max value, skewness, and kurtosis of depressive symptoms. The five highest means of the depressive symptoms were D05 (“I felt hopeful about the future”), D07 (“My sleep was restless”), D08 (“I was happy”), D01 (“I was bothered by things that do not usually bother me”) and D03 (“I felt depressed”), whereas D06 (“I felt fearful”) had the lowest mean.

**Table 2:** Descriptive statistics of the depressive symptoms (*n* = 1926).

Label	Abbreviation	Mean	*SD*	Min	Max	Skewness	Kurtosis
Bother	D01	0.95	1.09	0	3	0.72	−0.89
Mind	D02	0.90	1.08	0	3	0.81	−0.76
Depressed	D03	0.92	1.07	0	3	0.78	−0.78
Effort	D04	0.87	1.11	0	3	0.89	−0.73
Hopeful	D05	1.24	1.27	0	3	0.36	−1.57
Fearful	D06	0.36	0.80	0	3	2.19	3.63
Restless	D07	1.13	1.22	0	3	0.50	−1.38
Happy	D08	1.01	1.17	0	3	0.68	−1.08
Lonely	D09	0.56	0.98	0	3	1.53	0.92
Getgo	D10	0.38	0.83	0	3	2.17	3.47

### 3.2 Depressive symptom network

**Figure 1:**
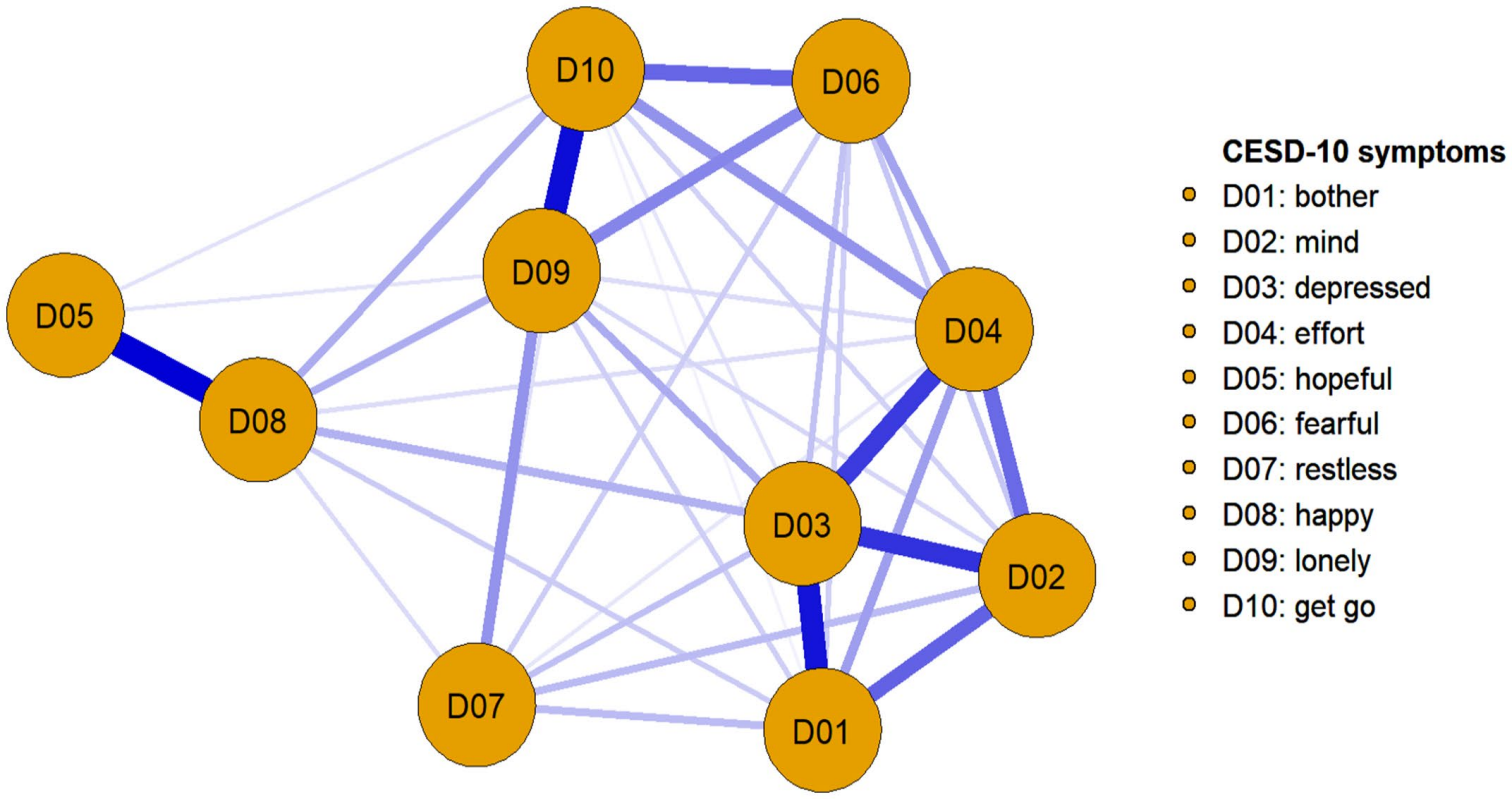
The 10-item depressive symptoms’ network structure of the migrant middle-aged and older adults. The thickness of edge denotes the association strength.

Figure 1 shows the network frameworks of 10 depressive symptoms-related items of the respondents. In general, all of the items were positively associated with one another within the network. In particular, strong connections emerged between D05 (“I felt hopeful about the future”) and D08 (“I was happy”) (r = 0.311), D09 (“I felt lonely”), and D10 (“I could not get going”) (*r* = 0.286), D01 (“I was bothered by things that do not usually bother me”), and D03 (“I felt depressed”) (*r* = 0.277), D02 (“I had trouble keeping my mind on what I was doing”), and D03 (“I felt depressed”) (*r* = 0.240), D03 (“I felt depressed”), and D04 (“I felt everything I did was an effort”) (*r* = 0.230).

The depressive symptom network’s centrality indexes of strength, closeness, and betweenness are shown in Table 3. For strength centrality, the five nodes with the highest strength values were D03 (“I felt depressed”), D09 (“I felt lonely”), D10 (“I could not get going”), D04 (“I felt everything I did was an effort”), and D02 (“I had trouble keeping my mind on what I was doing”). For closeness centrality, the five nodes with the highest closeness were D03 (“I felt depressed”), D09 (“I felt lonely”), D10 (“I could not get going”), D04 (“I felt everything I did was an effort”), and D02 (“I had trouble keeping my mind on what I was doing”). For betweenness centrality, the five nodes with the highest betweenness were D03 (“I felt depressed”), D08 (“I was happy”), D04 (“I felt everything I did was an effort”), D09 (“I felt lonely”), and D10 (“I could not get going”). The strength index was more reliable than other indexes (closeness and betweenness) (Robinaugh et al., 2014), and the most important symptom was selected by the value of the strength index. Therefore, the results found that D03 (“I felt depressed”) was the most important depressive symptom among the migrant middle-aged and older people. In general, the CS-coefficient of this network indicated that strength (CS = 0.750), closeness (CS = 0.672), and betweenness (CS = 0.283) all reached the cut-off value of 0.25. The estimated accuracy and stability for edge weights and node centrality showed that the network was relatively stable.

**Table 3:** Values of strength, closeness and betweenness.

Node	Strength	Betweenness	Closeness
D01 (bother)	0.298	−0.836	−0.134
D02 (mind)	**0.322**	−0.557	**0.160**
D03 (depressed)	**1.601**	**1.950**	**1.416**
D04 (effort)	**0.382**	**0.279**	**0.557**
D05 (hopeful)	−1.927	−0.836	−1.614
D06 (fearful)	−0.461	−0.836	−0.647
D07 (restless)	−1.196	−0.836	−1.227
D08 (happy)	−0.173	**1.393**	−0.459
D09 (lonely)	**0.728**	**0.279**	**1.001**
D10 (getgo)	**0.425**	**0.000**	**0.948**

*Z*-scores are displayed in the table rather than raw centrality indices for ease of interpretability. Bold values indicate the five nodes with the highest values.

### 3.3 Network estimation

**Figure 2:**
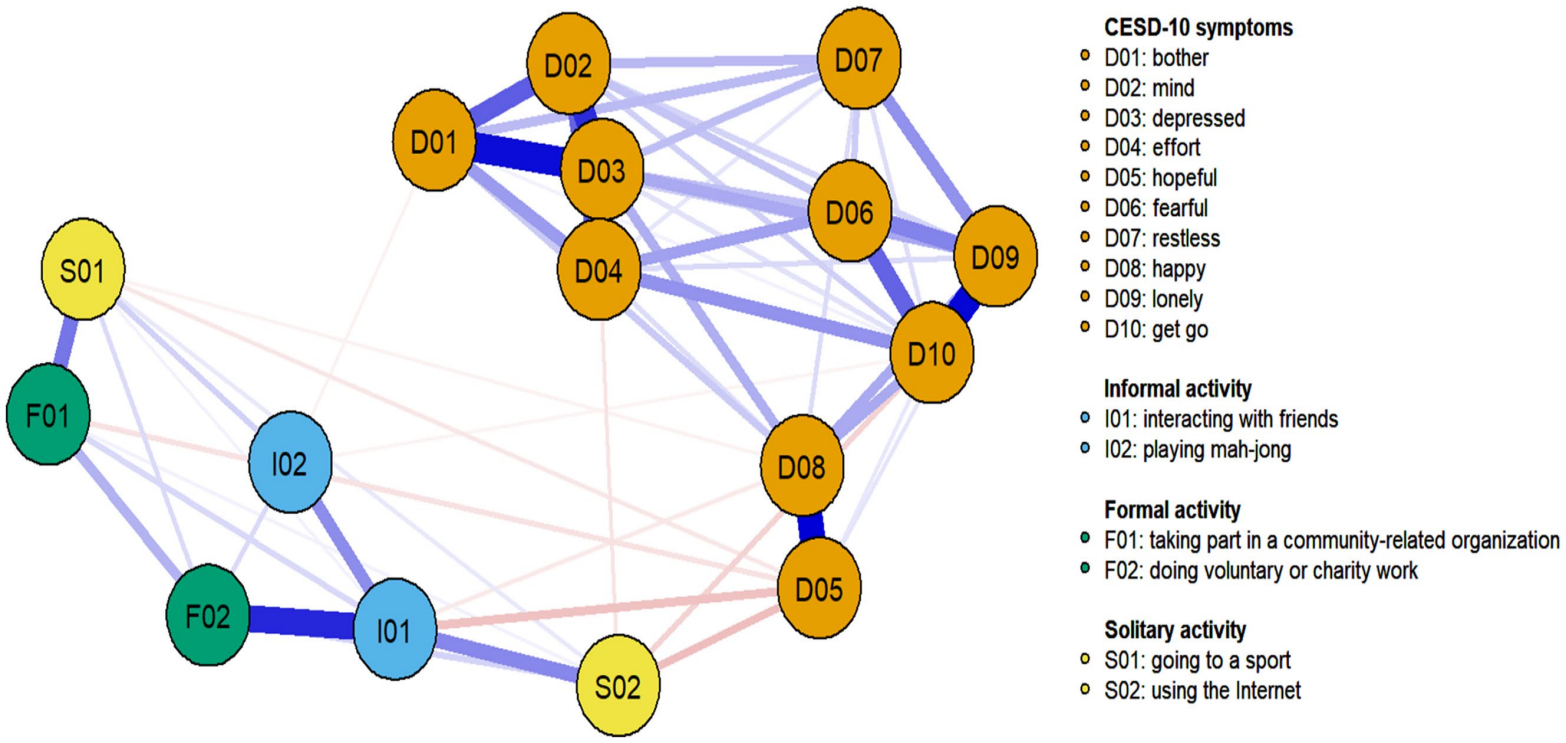
Partial correlation network of the combined set of depressive symptoms and social activities for the migrant middle-aged and older adults. Blue lines represent positive relationships, red lines negative ones, and the thickness of edge denotes the association strength.

Figure 2 shows the estimated networks with factors for the migrant middle-aged and older people. Overall, the associations between social activities and depressed symptoms were negative.

Interacting with friends (I01) was negatively associated with two depressive symptoms (sorted by decreasing partial correlation): D05 (*r* = −0.080) and D08 (*r* = −0.037). Playing mah-jong (I02) was negatively associated with two depressive symptoms (sorted by decreasing partial correlation): D01 (*r* = −0.035) and D10 (*r* = −0.032). Taking part in a community-related organization (F01) was negatively associated with D05 (*r* = −0.053). Going to a sport (S01) was negatively associated with two depressive symptoms (sorted by decreasing partial correlation): D05 (*r* = −0.040) and D08 (*r* = −0.033). Using the Internet (S02) was negatively associated with three depressive symptoms (sorted by decreasing partial correlation): D05 (*r* = −0.088), D10 (*r* = −0.062), and D04 (*r* = −0.046). The CS-coefficient indicated that strength (CS = 0.750) and closeness (CS = 0.439) were higher than the minimum values (0.25), while betweenness centrality (CS = 0.050) was unstable.

## 4 Discussion

Using network analysis, this study identified the central symptom and pathways of association with depressive symptoms. In addition, based on activity theory, this study also analyzed the specific pathways of informal, formal, and solitary activities on depressive symptoms. The study expand the understanding of the mechanisms of depressive symptom formation and development in migrant middle-aged and older adults and have potential to provide a new strategy of non-pharmacological interventions to alleviate depressive symptoms.

The current study indicated that the network structure of the migrant middle-aged and older adults depression indicated that D03 (“I felt depressed”) was the most important symptom, followed by D09 (“I felt lonely”), D10 (“I could not get going”), D04 (“I felt everything I did was an effort”), and D02 (“I had trouble keeping my mind on what I was doing”). Moreover, D03 (“I felt depressed”) had a strong relationship with D01 (*r* = 0.278), D02 (*r* = 0.240), and D04 (*r* = 0.230), suggesting that it was the most central symptom. Namely, the emergence of D03 probably activates other depression symptoms, resulting in a worsening of depression among the migrant middle-aged and older group. This result is not entirely consistent with the findings of existing studies. Murri et al. (2020) found that the core symptom of depressive symptoms in European older adults was “death wishes.” In this study, we found that “I felt depressed” was the critical depressive symptom among Chinese migrant middle-aged and older adults. This difference may be due to the unique characteristics of this group. Migrant middle-aged and older adults are a special group in the process of China’s urbanization. Due to the restriction of the urban–rural household registration system, they face various structural and social barriers in the city (Zhang et al., 2021), and their mental health is a matter of concern (Liu et al., 2020). Research findings revealed that in the treatment of depression among migrant middle-aged and older individuals in China, it is vitally important to pay attention to their depressed mood.

Regarding the factors, it turned out that different types of social activities seem to relieve different depressive symptoms. In terms of alleviating depression symptoms among Chinese middle-aged and older adults, informal activities are mainly expressed through positive emotions and somatic symptoms. The positive associations of interacting with friends (I01) on depressive symptoms in older adults were shown mainly through promoting positive emotions (D05 “I felt hopeful about the future” and D08 “I was happy”), which is consistent with existing research findings. The reason is that friendships characterized by reciprocity and being needed bring greater emotional satisfaction to older adults (Li et al., 2014). Older adults connected to friends are more likely to receive emotional support from friends and may be less lonely than their counterparts (Lee and Kim, 2014). In addition, the positive associations of playing mah-jong (I02) on depressive symptoms were mainly revealed through somatic symptoms (D01 “I was bothered by things that do not usually bother me” and D10 “I could not get going”). Playing mah-jong is a popular social recreation program for middle-aged and older adults. It can be viewed as a way for migrant middle-aged and older adults to spend their spare time and establish social relationships with neighbors in migrant places (Lee and Kim, 2014; Wang et al., 2020), which will be conducive to solving their daily bothers and continuing their lives in the city.

In terms of formal activities, positive associations between formal activities and depressive symptoms were primarily demonstrated through enhanced positive mood. The positive association between taking part in a community-related organization (F01) and depressive symptoms was primarily demonstrated through enhanced positive emotions (D05 “I felt hopeful about the future”). Studies have found that community-related organizations and activities provide older adults with social resources, strengthen their social interactions, and facilitate faster positive emotions from functional decline (Hao et al., 2017; Kim et al., 2017). In addition, Previous research has suggested that volunteering was associated with lower levels of depression (Musick and Wilson, 2003; Hong et al., 2009). However, our finding is inconsistent with a previous study that doing voluntary, or charity work (F02) was not associated with depressive symptoms. This inconsistency might be explained by several factors: Firstly, the majority of migrant middle-aged and older adults in China possess low educational levels, which could hinder their participation in volunteer activities, as evidenced by their minimal involvement in such efforts (Shibin et al., 2015). Secondly, the residential registration system often excludes or limits these individuals from joining many community-organized volunteer activities (Hu and Li, 2020). Consequently, the potential of volunteer activities to reduce depressive symptoms in this group is somewhat constrained. Our findings expand on previous understanding by showing that the mental health benefits of volunteer activities for middle-aged and older adults can vary significantly depending on the social context.

As for solitary activities, the association between solitary activity and depression was primarily demonstrated through positive mood and somatic symptoms. The positive associations between going to a sport were primarily manifested through positive emotions (D05 “I felt hopeful about the future” and D08 “I was happy”). Research has found that playing sports may increase positive emotions and be associated with a more “positive outlook.” The neurochemical mediators of positive mood produced by playing sports may interest with underlying stress protection, favoring improvements in the consequences of stress as well as reducing unpleasant emotional states (Greenwood, 2019). In addition, the positive associations between using the Internet (S02) and depressive symptoms were mainly manifested through somatic symptoms (D04 “I felt everything I did was an effort” and D10 “I could not get going”) and positive emotions (D05 “I felt hopeful about the future”). On the one hand, using the Internet does require a certain amount of brain activity, and middle-aged and older adults can mitigate the functional decline that comes with aging. On the other hand, the Internet can overcome social and spatial constraints, especially for migrant middle-aged and older adults, who can keep communicating with family and friends and maintain an extensive social network through the Internet (Aggarwal et al., 2020; Liu et al., 2020). This facilitates their access to emotional support and positive emotional values. Consequently, it is also an essential method for preventing a decline in mental health among middle-aged and older migrants.

In this study, we found that informal and solitary activities are negatively associated with more depressive symptoms, whereas formal activities are negatively linked with the least symptoms. These results were inconsistent with the activity theory (Lemon et al., 1972) and many existing prior studies (Belvederi Murri et al., 2020; Kim et al., 2021). This inconsistency may lie in the fact that the social context and personal characters of middle-aged and older migrants in China is different from that of Western and Asian older people. In China, after middle-aged and older migrants enter a strange habitat from their initial domiciles, they are often confronted with many institutional barriers due to the residential registration system and forced to build up their new social network since they break away from their original social relations (Liu et al., 2020). What’s more, both significantly impact which type of social activities may negatively be associated with depressive symptoms among middle-aged and older migrants. For this, future studies on the application of the activities theory need to consider the local context and the sample characters. Besides that, this paper also indicated that people with varying activity tendencies could benefit from several kinds of activities rather than a single particular activity type. For instance, this study highlighted that most activities are negatively associated with positive emotion (e.g., hopefulness), though it was not the central depressive symptom.

This study utilized network analysis approach to deeply analyze the association pathways of depressive symptoms among mobile middle-aged and older adults in China as well as to unveil the specific association pathways of informal, formal, and solitary activities on depressive symptoms, enriching the exploration of the relationship between social activities and depressive symptoms in the Chinese context. However, several limitations of this current study should be noted. First, analyses were based on cross-sectional data, which prevented us from determining causality and temporality between different types of social activities and depressive symptoms. Second, this study used the 10-item Center for Epidemiologic Studies-Depression scale to measure depression. According to the DSM-5, its diagnostic function is relatively inferior as a screening tool; interpretation of the results should be done with caution. In addition, the resulting network structure could be changed when modified contents or different measures are used. Therefore, future studies are encouraged to duplicate this study, which is valuable for testing and widening the investigation of this paper. Last but not least, in network theory, the number of relationships between factors and depressive symptoms asserted the preferred importance of these factors; their positions could be changed as the contents are enriched.

## 5 Conclusion

This current research revealed that depressed mood showed the highest value of centrality among migrant middle-aged and older adults in China. In terms of alleviating depressive symptoms among Chinese migrant middle-aged and older adults, informal activities were mainly manifested through positive emotions and somatic symptoms. Formal activities were mainly revealed through positive emotions. Solitary activities were manifested primarily through positive emotions and somatic symptoms. In addition, our study suggested that informal and solitary activities exert a stronger association than formal activities in reconciling depression among middle-aged and older migrants.

## Data availability statement

Publicly available datasets were analyzed in this study. This data can be found here: https://charls.pku.edu.cn/.

## Ethics statement

This survey obtained ethical approval from the Biomedical Ethics Review Committee of Peking University (approval number: IRB00001052-11015). The studies were conducted in accordance with the local legislation and institutional requirements. The participants provided their written informed consent to participate in this study.

## Author contributions

QL: Conceptualization, Data curation, Formal analysis, Funding acquisition, Investigation, Methodology, Project administration, Resources, Software, Supervision, Validation, Visualization, Writing – original draft, Writing – review & editing. YW: Data curation, Formal analysis, Methodology, Visualization, Writing – original draft, Writing – review & editing. CY: Writing – original draft, Writing – review & editing, Conceptualization. YP: Supervision, Writing – original draft, Writing – review & editing.
