# Supplementary material for: Social activities and depressive symptoms among migrant middle-aged and older adults in China: a network analysis
Source: Front Psychol. 2024 Jun 13;15:1376180. doi: 10.3389/fpsyg.2024.1376180 (PMC11210719; doi:10.3389/fpsyg.2024.1376180)
Supplement: Supplementary file 1 [file Data_Sheet_1.doc]

**Appendix A**

Appendix A: Full information about the 10-item Center for Epidemiologic Studies Depression Scale

| Item | Content | Label |
| --- | --- | --- |
| 1 | I was bothered by things that don’t usually bother me | bother |
| 2 | I had trouble keeping my mind on what I was doing | mind |
| 3 | I felt depressed | depressed |
| 4 | I felt everything I did was an effort | effort |
| 5 | I felt hopeful about the future | hopeful |
| 6 | I felt fearful | fearful |
| 7 | My sleep was restless | restless |
| 8 | I was happy | happy |
| 9 | I felt lonely | lonely |
| 10 | I could not get “going” | getgo |

Appendix B


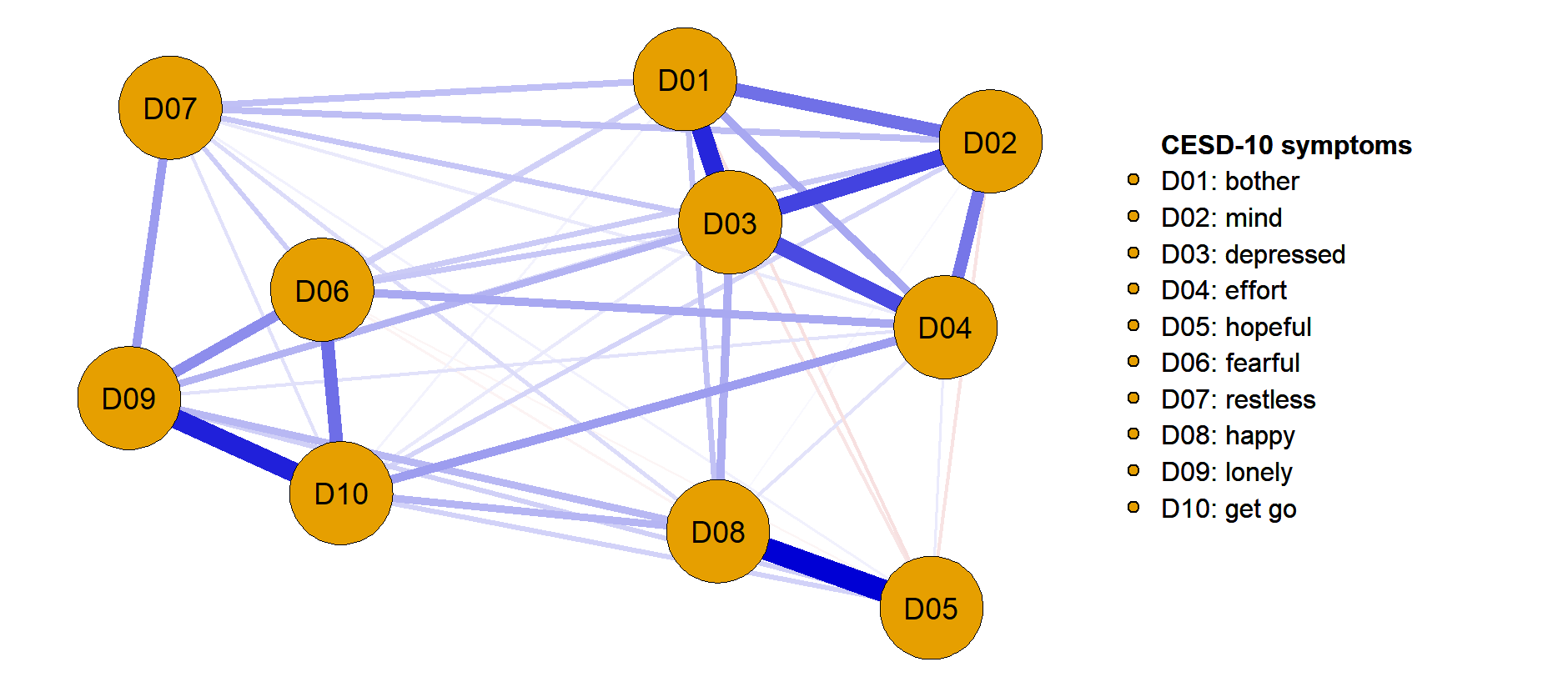


Figure S1. The 10-item depressive symptoms’ network structure of the migrant middle-aged and older adults by using non-regularized partial-correlation. The thickness of edge denotes the association strength.


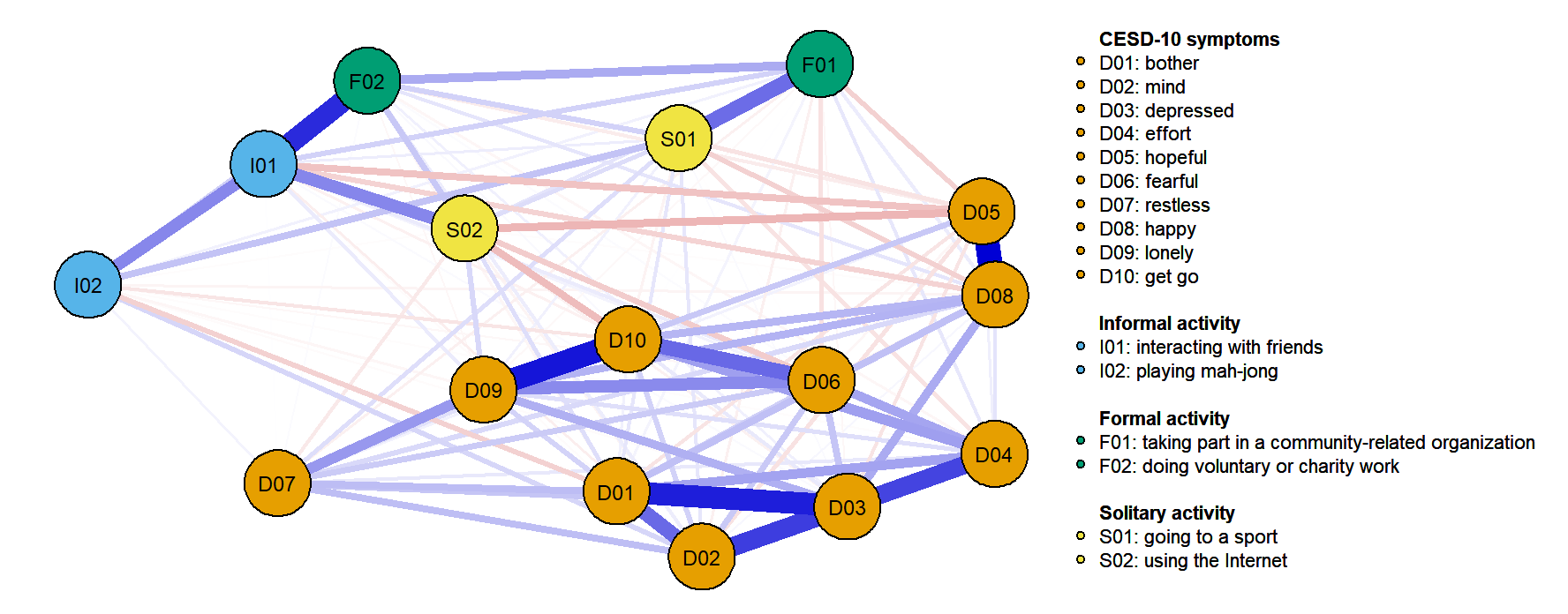


Figure S2. Non-regularized partial correlation network of the combined set of depressive symptoms and social activities for the migrant middle-aged and older adults. Blue lines represent positive relationships, red lines negative ones, and the thickness of edge denotes the association strength.


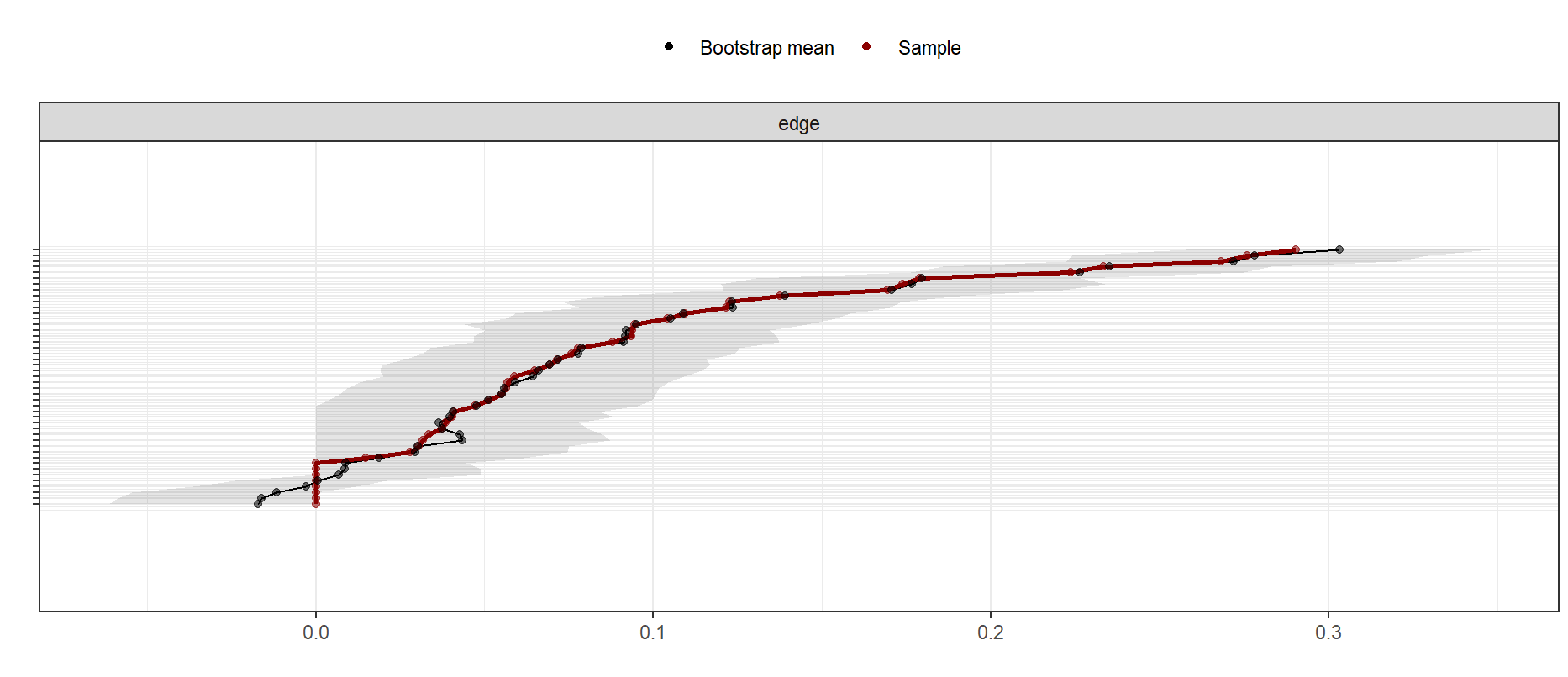


Figure S3. Bootstrapped confidence intervals of estimated edge-weights within the structure network of depressive symptoms. The red line indicates the sample values and the gray area the bootstrapped CIs. Each horizontal line represents one edge of the network from the highest edge-weight to the lowest edge-weight.


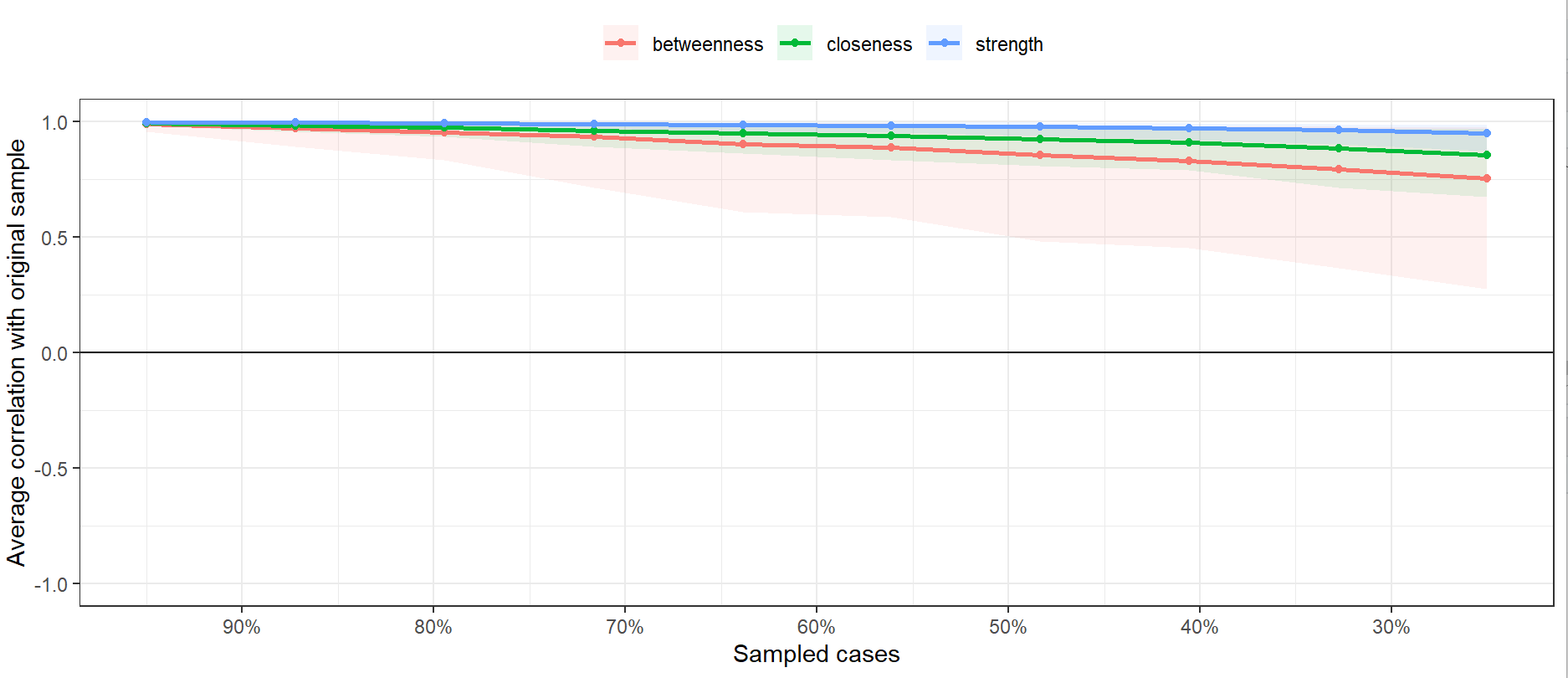


Figure S4. Average correlations of the node strength centrality estimates obtained from the structure network of depressive symptoms. Lines indicate the means, and areas indicate the range from the 2.5th quantile to the 97.5th quantile.


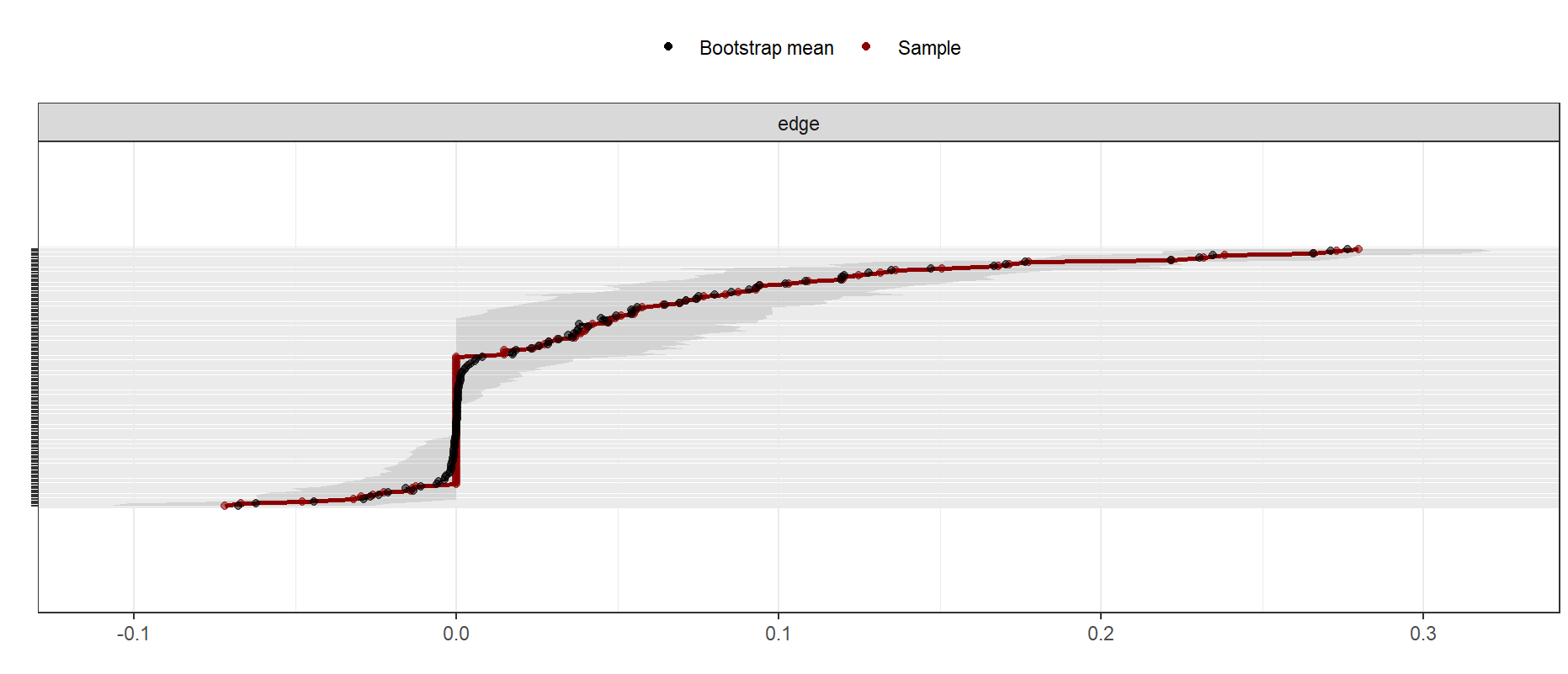


Figure S5. Bootstrapped confidence intervals of estimated edge-weights within the structure network of depression, informal activities, formal activities and solitary activities. The red line indicates the sample values and the gray area the bootstrapped CIs. Each horizontal line represents one edge of the network from the highest edge-weight to the lowest edge-weight.


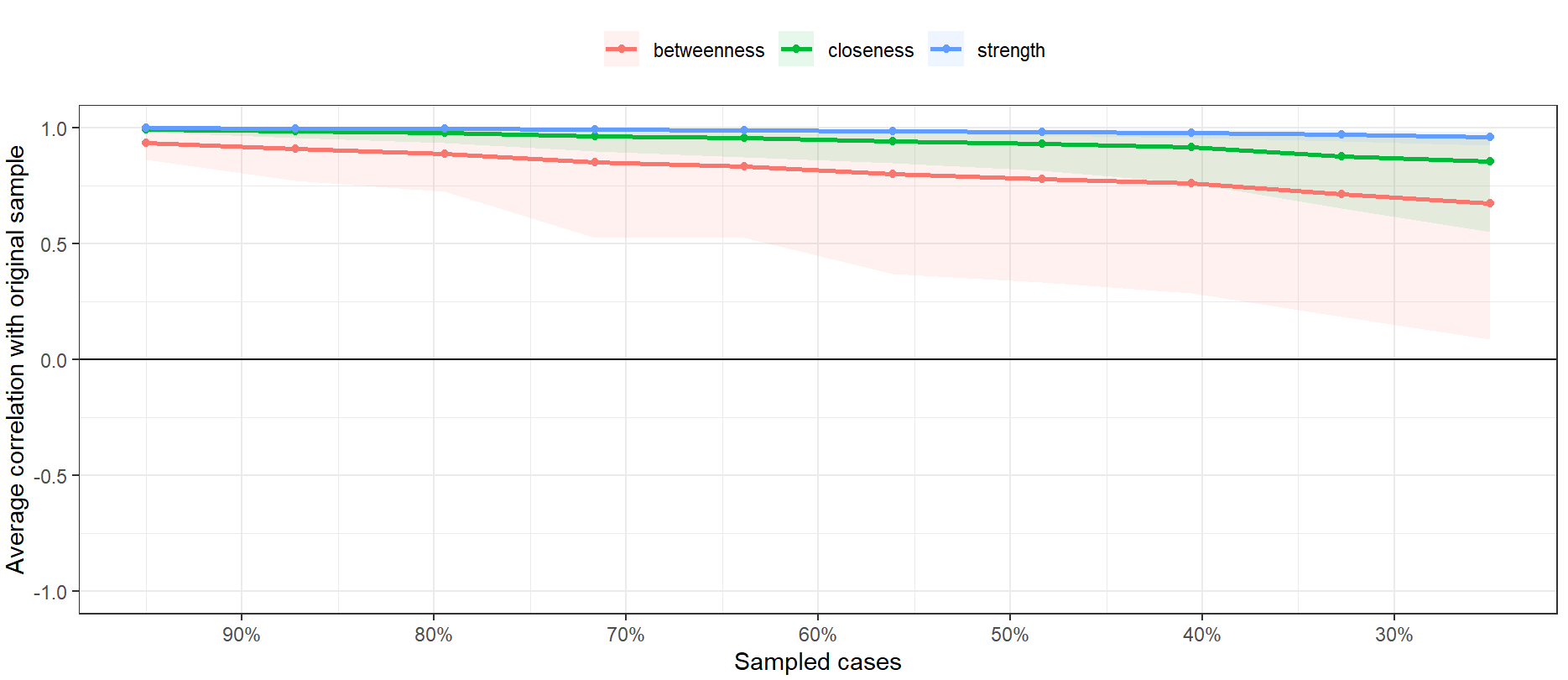


Figure S6. Average correlations of the node strength centrality estimates obtained from the structure network of depression, informal activities, formal activities and solitary activities. Lines indicate the means, and areas indicate the range from the 2.5th quantile to the 97.5th quantile.
